# Advancing the application of systems thinking in health: South African examples of a leadership of sensemaking for primary health care

**DOI:** 10.1186/1478-4505-12-30

**Published:** 2014-06-16

**Authors:** Lucy Gilson, Soraya Elloker, Patti Olckers, Uta Lehmann

**Affiliations:** 1 School of Public Health and Family Medicine, University of Cape Town, Anzio Road, Observatory, Cape Town 7708, South Africa; 2 Department of Global Health and Development, London School of Hygiene and Tropical Medicine, Keppel Street, London WC1E 7HT, UK; 3 City of Cape Town Department of Health, Mitchell’s Plain health sub-district, Park Avenue, Mitchell’s Plain 7785, South Africa; 4 Western Cape Department of Health, Klipfontein/Mitchells Plain Sub-structure Office, Lentegeur Hospital, Highlands Drive, Lentegeur, Mitchells Plain 7785, South Africa; 5 School of Public Health, University of the Western Cape, Robert Sobukwe Road, Bellville 7535, South Africa

**Keywords:** Discretionary power, Complex adaptive systems, Front line workers, Leadership, Primary health care, Sensemaking

## Abstract

**Background:**

New forms of leadership are required to bring about the fundamental health system changes demanded by primary health care (PHC). Using theory about complex adaptive systems and policy implementation, this paper considers how actors’ sensemaking and the exercise of discretionary power currently combine to challenge PHC re-orientation in the South African health system; and provides examples of leadership practices that promote sensemaking and power use in support of PHC.

**Methods:**

The paper draws on observational, interview, and reflective data collected as part of the District Innovation and Action Learning for Health Systems Development (DIALHS) project being implemented in Cape Town, South Africa. Undertaken collaboratively between health managers and researchers, the project is implemented through cycles of action-learning, including systematic reflection and synthesis. It includes a particular focus on how local health managers can better support front line facility managers in strengthening PHC.

**Results:**

The results illuminate how the collective understandings of staff working at the primary level - of their working environment and changes within it – act as a barrier to centrally-led initiatives to strengthen PHC. Staff often fail to take ownership of such initiatives and experience them as disempowering. Local area managers, located between the centre and the service frontline, have a vital role to play in providing a leadership of sensemaking to mediate these challenges. Founded on personal values, such leadership entails, for example, efforts to nurture PHC-aligned values and mind-sets among staff; build relationships and support the development of shared meanings about change; instil a culture of collective inquiry and mutual accountability; and role-model management practices, including using language to signal meaning.

**Conclusions:**

PHC will only become a lived reality within the South African health system when frontline staff are able to make sense of policy intentions and incorporate them into their everyday routines and practices. This requires a leadership of sensemaking that enables front line staff to exercise their collective discretionary power in strengthening PHC. We hope this theoretically-framed analysis of one set of experiences stimulates wider thinking about the leadership needed to sustain primary health care in other settings.

## Background

Reform and renewal are fundamental features of every health system, though the ambition and scale of change varies over time and between countries. Twenty years after the election of its first democratic government, South Africa continues to strive for an improved health system – a health system that better meets the needs and preferences for treatment, care, and dignity, of all its population. The fragmented health system inherited from the previous era, with multiple organizational structures, levels, and programmes, was shaped by the perverse political and economic goals of the Apartheid state [1]. Various policy, organizational, and resource allocation reforms have been implemented since 1994 to re-orient the system towards population health need and equity goals. Nonetheless, recent reviews have highlighted slow progress, particularly in establishing a functional district health system (DHS) as a basis for strengthening primary health care (PHC) [2,3]. Towards Universal Health Coverage, and in line with global policy directions [4,5], South Africa has, therefore, placed renewed urgency on PHC and DHS development [6-8].

International experience shows that re-orienting health systems towards PHC challenges existing ways of working [4,9]. In South Africa, dispersed accountability, complex rules and procedures, and an organizational culture of deference to hierarchy within it also “*overwhelm rational policy debate and the implementation of new policy*” [10]. As a result, and as pointed out by the National Department of Health, the South African health system remains strongly hospi-centric and specialized, with decision-making driven more by service than population needs [6]. As elsewhere, the pro-active pursuit of population health needs and equity goals in PHC strengthening requires, therefore, fundamental changes in the way health system actors think and work, in its organizational culture, supported by new forms of health system leadership [11,12]. Although there is only limited evidence about what such leadership entails, theoretical perspectives suggest that ‘reculturing an organisation’ involves empowering front line workers to think and work differently by encouraging subtle change in the values, customs, relationships, and conversations shaping their behaviour [13-15].

In this paper, we present experience to illuminate both the challenges that confront efforts to strengthen PHC within the South African health system, and the nature of leadership needed to mediate such organizational change. Our analysis is framed by the concepts of sensemaking and discretionary power, drawn from theory on complex adaptive systems and policy implementation, respectively. We argue that, to become a lived reality within the DHS, those working to support primary and community-based services, including PHC facility managers and their staff, must be able to make sense of PHC-promoting policies and plans, and incorporate them into their everyday routines and practices. This requires new forms of leadership by the health system’s middle managers, namely sub-district managers: a leadership of sensemaking in support of PHC strengthening.

We are a team of health system managers and researchers working together to understand and act in the district health system, through cycles of collaborative action and learning, in Mitchell’s Plain health sub-district, Cape Town, South Africa. Our analysis represents a theoretically-framed reading of one particular set of experiences in one particular place, generated through a careful, systematic, and reflective research collaboration. We do not seek to derive discrete policy lessons about particular activities that can strengthen PHC in South Africa or elsewhere. Instead, recognizing policy learning as an organic process [16], our intention is to stimulate those working in other settings to think differently about the forms of leadership needed to sustain PHC.

## Methods

### Study approach and focus, data collection, and data analysis

The experiences we present are drawn from the District Innovation and Action Learning for Health Systems Development (DIALHS) project, initiated in 2010 as a service-research partnership focused on governance issues within the Mitchell’s Plain health sub-district, Cape Town. It involves collaboration between two health authorities (the City of Cape Town and the provincial health department of the Western Cape government), and two universities (the Universities of Cape Town and the Western Cape).

**Figure 1:**
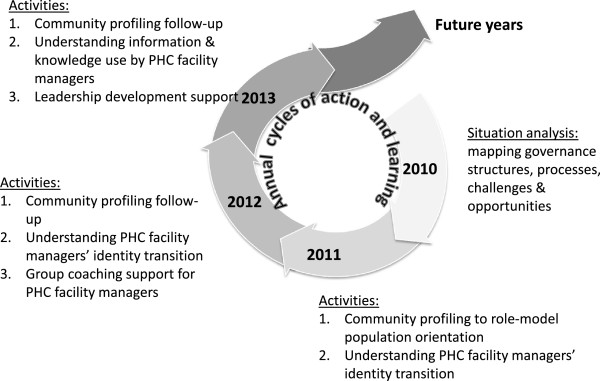
The DIALHS cycles of collaborative action and learning.

We began our engagement by conducting a situation analysis to understand the managerial structures and processes of the sub-district and its location in broader district functioning. Discussion of this analysis then led us to consider further how sub-district managers can better support PHC facility managers to lead their staff teams. As Figure 1 indicates, we have subsequently addressed this concern through iterative cycles of collaborative action and learning [17,18], including a focus on facility managers’ experience, that have entailed cycles of data collection, analysis, and interpretation. Reflective practice has been a common approach in all our activities, and is itself an intervention in managerial practice [19].

Table 1 outlines the range of data generated across our activities, which include transcripts and notes of general reflective discussions within the research team, and with managers. This paper is based specifically on the internal report of the initial situation analysis (2010), notes of key DIALHS discussions within sub-district management meetings (2010–2012), researcher field notes (2010–2012), transcripts and notes of 22 interviews and personal meetings with managers at district and sub-district level (2010–2013), and transcripts of 5 research team reflective discussions (2011–2012). Following the principles of thematic analysis, these data were initially systematically reviewed with our conceptual starting points in mind (see below), by the researcher-authors (LG, UL). An initial synthesis and narrative of experience was then developed for discussion with the other authors, the two primary sub-district managers (SE, PO), in a validation process equivalent to member and peer-checking that also generated further insights drawn into the final paper. Therefore, in our work, we have been systematic, a core criterion of validity in action learning [17], as in other research [20]. We have also allowed double-loop learning [21], deepening understanding of experience through reflection during our work and in developing this paper – stepping back from the initial narrative, interrogating our assumptions, and viewing it from different perspectives.

**Table 1:** Data generated within DIAHLS project 2010–2013

**Activities and engagements which generated data**		**Data generated**	
***Situational analysis***		Review of policy documents and minutes of statutory meetings	
		Stakeholder interviews	
		Observations of meetings	
***Cycles of***	***Planning interventions***	Presentations and meeting/workshop notes; document reviews	Composite reports
	Community profiling and local area groups		
	Support for environmental health practitioners		
	Support for PHC facility managers		
	HIV/AIDS & TB programme roles		
	***Implementing of interventions***	Presentations, notes of meetings, field notes, and reports	
	***Review and reflection***	Notes of meetings with teams involved in intervention	
		Presentations and reports to ISDMT meetings	
***Research sub-studies***			
The transition process from nurse to facility manager		Interviews	
The information used by facility managers in routine decision-making		Observations	
***Meetings and reflections of research team***		Transcriptions and notes of reflective meetings of research team	
***Interviews and reflective conversations with sub-district and district managers***		Notes of meetings with district managers	
		Notes of meetings with sub-district managers	

Our work has ethical clearance from the Human Research Ethics Committee of the Faculty of Health Sciences, University of Cape Town (Ref 039/2010), and research approval from both the City of Cape Town and Western Cape Provincial Government Department of Health.

### Conceptual foundations

Sensemaking can be understood as “*the process individuals undertake as they try to understand what is going on around them, as they try to make sense of events and experiences*” [22]. In sensemaking, our mental models, that is our beliefs and assumptions about how the world works [23,24], help us to notice phenomena in our environment, which we then categorise and label, making meaning of them, ultimately as a basis for acting. Sensemaking is, therefore, about the interplay of interpretation and action [25-27].

The adaptive agents within complex adaptive systems (CAS) are sensemakers, whose interpretations of their world are shaped by the system paradigm, the underpinning, often unspoken but shared, social agreements about the nature of reality in that system [23]. Because agents in every system are interconnected and interdependent, their many daily interactions also result in the emergence of shared ways of being and doing, patterns of collective behaviour that are taken for granted [28]. These system structures, the manner in which a system’s elements are organized, include, for example, practices of inter-personal engagement and information flows; they, in turn, shape the patterns of organizational life that generate the events that we most easily notice [24].

Although not recognized as CAS theory, Lipsky’s [29,30] theory of Street Level Bureaucracy (SLB) illuminates CAS ideas with specific reference to public policy implementation. The discretionary power of ‘street level’, or front line, workers in public bureaucracies exists because they are “*free to make a choice among possible courses of action and inaction*” [31] within the rules shaping their behaviour, allowing them to translate policy through their practices and interactions with clients. Lipsky argued that as they interpret, choose, and act, they are guided by the mental models they develop to manage their demanding work settings characterized by heavy workloads, resource constraints, and centrally directed and often unclear, policy imperatives. In these settings the unanticipated consequences of the ways they manage their time and engage with their clients include limiting access to public services and treating clients disrespectfully. However, front line workers can also engage positively with clients, particularly when encouraged to use their discretionary power to be responsive to clients [32].

These bodies of theory both suggest that hierarchical, command, and control leadership practices do not take account of the reality of complex adaptive systems and policy implementation. SLB theory specifically notes that top-down action to control the use of discretionary power will only encourage front line workers to stereotype and disregard client needs [29]. The sensemaking literature suggests, moreover, that during times of organizational change individuals try to make sense of their experience by engaging with others, generating shared interpretations that, in turn, shape their behaviours and trigger further sensemaking. This cycle generates new, shared ways of working that may not be aligned with the intentions of new initiatives [26]. The theory suggests, therefore, that particular forms of leadership are required to implement policy and bring about organizational change in CAS. Such leadership needs to be distributed across all levels of an organization, placing particular demands on middle-level managers [33]. Moreover, as “*real leverage exists deep in the recesses of the systems – mind-sets, values, beliefs – where identify is created*” [28], such leadership needs to mediate sensemaking and support changes in the shared assumptions about how people should act (so influencing their exercise their discretionary power) in different situations [23,24].

## Results

Drawing on our conceptual foundations we now present experience from a health sub-district in Cape Town, considering both sensemaking and exercises of discretionary power, and leadership practices that seek to recognize both in supporting new ways of working. We start by describing the setting of our work.

### Mitchell’s Plain health sub-district

In 1976, residents from 250 different communities across Cape Town were forcibly removed and settled in Mitchell’s Plain, when it was designated a ‘separate’ area for so-called ‘coloured people’. This birth in violent social dislocation reverberates to this day, and it is now one of the poorest areas of the city. High unemployment and low labour absorption (at 24% and 46%, respectively, according to the 2011 census) [34], as well as substance abuse and poor schooling, exacerbated by massive population growth, contribute to vicious cycles of poverty, crime, and social destabilization.

Public PHC services in Mitchell’s Plain health sub-district are provided to a population of over 510,000 (2011/2012) by both local and provincial government facilities focused, respectively, on preventive, promotive, and curative child health services, and adult curative care.^a^ Indicators point to a relatively strong public health service performance in the sub-district: a tuberculosis (TB) cure rate of 88%, immunization coverage at 93%, and 56% of ante-natal care visits occurring before 20 weeks. However, there are still numerous under-served communities, particularly in the newer, fast developing areas of the sub-district; and there are regular patient complaints about poor quality of care and areas of service delivery weakness. Key health problems include a co-infection rate of TB and HIV of 50%, non-communicable diseases, mental ill-health, and violence ([35], and data from 2011/2012 District Health Expenditure Review for Cape Town).

Efforts to integrate and strengthen PHC service provision across the two health authorities over the last 20 years have been complicated and hampered by a range of legal, labour, and financial obstacles. The 2008 establishment of the Metro District Health System (MDHS), as part of the provincial government’s wider vision of strengthening PHC,^b^ provided the structural platform for delivering comprehensive and integrated PHC services in Cape Town. At present, the Mitchell’s Plain integrated sub-district management team (ISDMT) coordinates service delivery in agreed areas between local (City of Cape Town, CoCT) and provincial government (MDHS).

**Figure 2:**
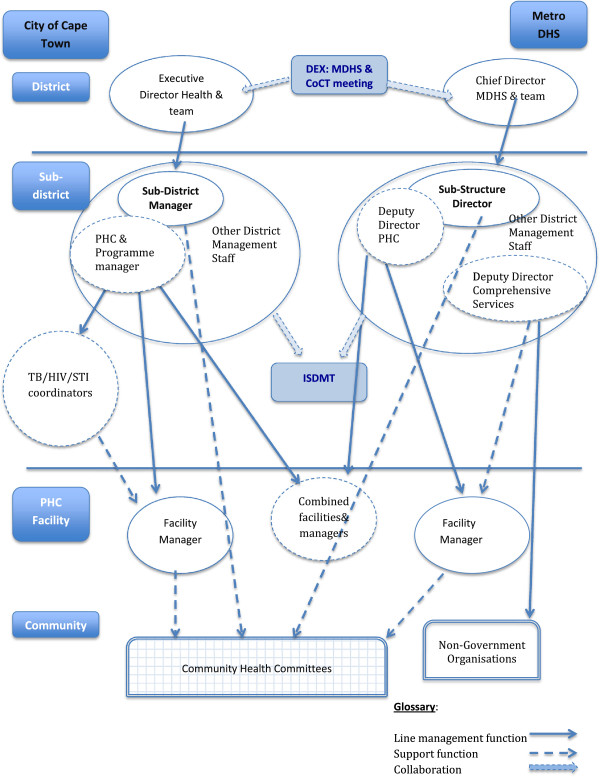
**Lines of authority in the Cape Town Metropolitan Health District [adapted from [**[35]**]].**

The two Mitchell’s Plain health managers were appointed to their current posts in 2005 (SE, CoCT sub-district manager) and 2012 (PO, MDHS sub-structure manager). Their position within the complex lines of authority that make up the Cape Town health system is shown in Figure 2. Located at the interface between top-down strategic planning processes and bottom-up operational decision-making processes and action, they are the middle managers [36] tasked with leading the establishment of a health system oriented towards population health needs as envisioned by provincial health policy (manager interview notes, 1 July 2010). Working within centrally-set budget limits and human resource management guidelines, they both have some decision-making latitude. Overall, they are responsible for the management of resources, people and perceptions (manager interview notes, 19th July 2010), and judge that their biggest challenges lie in managing people and their perceptions [35].

The sub-district health managers see PHC facility managers as key players in strengthening PHC in Mitchell’s Plain. These managers and their staff represent the face of the health system to the public, are responsible for its responsiveness to people and patients, and should be among the first to pick up community health needs and concerns.

### Mind-set challenges to PHC in Mitchell’s Plain

Over the last 20 years, PHC in Mitchell’s Plain has become “*a more complex environment for those working in and for the community. Nurses see a lot of sick children and sometimes the children die. Nurses don’t all necessarily have the right skills (for these situations, and given high staff turnover). So facility managers have to manage this complexity and also don’t all have the skills. And they have little confidence in the system – for example, ambulance services don’t arrive on time. And the managers don’t know how to talk to the community for example over deaths in facilities, or how to help staff cope with the demands*” (Manager interview notes, 13th April 2012).

In this environment, there is a duality of sensemaking and action. As a group, front line staff often seem to end up working against PHC-oriented change, even though as individuals they might support its goals. Facility managers and their staff commonly maintain collective mind-sets that are steeped in the autocratic and procedural cultures of a previous era and that run counter to a population health and PHC orientation. The sub-district managers note that some staff display rather “*authoritarian and autocratic attitudes towards patients, and do not want to share power with them*” (ISDMT notes, 19th Jan 2011). They also think that facility managers do not always “*understand the ‘big picture’ of facility services, that is, the health needs of the population they serve, the challenges patients face in in accessing services, and so the importance of new community-based activities and programmes. They don’t generate and use information to understand the needs of the population they serve, and ‘they don’t think beyond the people coming into the facility*” (Researcher field notes, 6th July 2012). Indeed, as the PHC facility managers are all professional nurses by training and receive limited support when first appointed, many of them prefer, and even feel more confident, in their clinical rather than managerial roles [37].

Often, facility managers also seem to work in a very procedural way – adopting an attitude that says, “*I want the piece of paper that tells me how to act*” (Research team notes, 14th December 2010). Whilst recognizing that these managers are mostly quite capable and competent, sub-district managers often see them as having an “*external locus of control*” –being too passive, not persistent in dealing with problems in their facilities, including complaints from patients, or in keeping track of their own performance (Manager interview notes, various dates; Research meeting notes, 4th December 2012). Timekeeping and keeping up with routine schedules of activities is also judged to be poor. When given new decision-making authority, facility managers appear to respond fearfully rather than by embracing the opportunity: it seems as if “*they don’t take responsibility for new activities or actions to improve services. They say to their staff, ‘the sub-district manager said you must do it’, rather than projecting a positive image of the activity and owning it*” (Researcher field notes, 6th July 2012). Such resistance is typical of the exercise of discretionary power by street level bureaucrats [29], but also reflects ‘change fatigue’ [38-40] and organizational uncertainty after nearly 20 years of constant health system change. The continuing debates about if, when, and how local and provincial government PHC services will be integrated in Cape Town have only aggravated this uncertainty.

The retention of paternalistic and autocratic approaches is, moreover, not restricted to frontline workers. Staff survey results indicate that communication practices are commonly perceived to be quite hierarchical in the wider health system [41]. Some sub-district managers even talk about PHC facility managers as “*their children*”, expressing the need “*to watch over them*” (Research meeting notes, 4th December 2012). An ‘ambivalence towards authority’ among South Africa civil servants is, moreover, a general apartheid legacy [42] that is compounded, within the health system, by “*the accumulated weight of existing practices and procedures, together with embedded hierarchies that institutionalise a specific distribution of power and privilege*” [10].

### Sensemaking and resistance to centrally-led PHC improvement targets

In strengthening PHC, the experience around annual targets provides an important example of how sensemaking plays out, given dominant mind-sets. In line with provincial and local government health plans, annual targets are centrally established within strategic planning processes to drive PHC service improvements in Cape Town (e.g., for tuberculosis cure rates or extension of basic ante-natal care across facilities). These targets are backed up by regular monitoring through ‘plan, do, review’ (PDR) meetings where managers at different levels come together to examine facility, sub-district and district performance against targets, identify challenges and develop actions to address them.

Mid and senior health managers see these processes as “*providing standardized frameworks to guide lower level managers and, more specifically, providers, to work differently to better meet population health needs*” (Manager interview notes, 19th July 2010). They also argue that “*policy provides a stable structure within which people know what is expected of them*” (Manager interview notes, 13th April 2012) and that standardization higher up the system is “*to give some predictability/logic to allow for innovation lower down… to bring the certainty needed for innovation lower down the system*” (Manager interview notes, 19th July 2010).

However, both positive and negative meanings have become attached to the word ‘targets’ in the Mitchell’s Plain health system discourse. The positive potential is expressed as “*directing people towards common goals, or giving people a motivating force*”. Reaching a target can, thus, bring a sense of achievement and positive energy (Research meeting notes, 4th December 2012). In contrast, the dominant language PHC facility managers and staff use around targets is quite negative – with targets seen as disempowering, as a disciplinary tool, and as encouraging or enabling micro-management by higher-level managers. Perhaps refracted through the prism of history and wider organizational culture, facility managers seem to understand the word ‘targets’ as authoritarian and therefore illegitimate: “*It’s all that is bad in the system… it also says ‘we don’t have agency’…‘we are bombarded, can’t do anything else’, so it removes accountability and responsibility for anything other than the target*” and so “*a lot of the target conversation is completely disembodied, it’s removed from the actual meeting of service needs”* (Research meeting notes, 5th December 2012).

Given the prevailing mental models, targets and the PDR processes are, in a sense, filtered through a power battle between managers seeking to give clear and consistent direction for strengthening PHC to multiple actors, and front line staff, shaped by histories and cultures of passivity and dominance, resisting change and afraid to take on new responsibilities. Although facility managers mostly comply with reporting requirements, they have not yet adopted the broader problem-solving attitude or willingness to take ownership of, and make effective, efforts to improve PHC services. As noted in other settings [29,43], central efforts, such as target setting, that seek both to contain the discretionary power of street level bureaucrats and direct it towards imposed goals often have unexpected consequences. Planned (imposed) change may encourage compliance without conviction [44], because, as sensemaking theory notes, it fails to provide spaces for the new forms of sensemaking necessary to support the intended changes [26]. Ultimately, therefore “*change is itself an interpretive process*” in which “[t]*he meaning of top down initiatives emerges bottom up*” [33].

### Supporting PHC strengthening through a ‘leadership of sensemaking’

From our first engagements, sub-district managers recognized the challenge of having to address the passivity of PHC facility managers and their staff, and their role in empowering them “*to work differently to meet needs*”, as critical issues in Mitchell’s Plain (Manager interview notes, 19th July 2010). Within the DIALHS collaboration we have subsequently, in a mixture of deliberate and spontaneous actions, tried out different ways of engaging and supporting PHC facility managers to recognize and address population health needs, working with their staff. Our initiatives include both new, joint activities (e.g., the community profiling initiative) and adaptations to routine processes and practices (e.g., the key performance area (KPA) process). All seek to encourage PHC facility managers to take ownership of their own performance, and that of their staff, as well as to hold them to account for it (Manager interview notes, 15th Oct 2012; 7th December 2012; 9th April 2013).

*The community profiling initiative (generating local knowledge, priorities, and action through multi-actor engagements)*

In early 2011, we initiated an activity that aimed to strengthen three inter-related planning and management priorities in the sub-district: i) ‘shifting the lens’ of service providers from a patient to a stronger population orientation in health system organization and functioning, as advocated by provincial and national policy guidelines; ii) moving the sub-district’s thinking and visioning beyond one-year planning cycles; and iii) strengthening relationships between service providers and community representatives.

Provincial and district management had recently emphasized the need to address health of populations, rather than patients alone, in their policy and planning guidelines. Yet, facility managers had repeatedly expressed uncertainty and frustration with the fact that they “did not know the communities they were serving”, did not know how to engage with other role players or access other health resources in communities, and were overwhelmed with the need to service short-term targets instead of being responsive to local priorities and needs.

A series of larger and smaller workshops brought together multiple stakeholders from health authorities and civil society to identify and map health resources and gaps, as well as to identify appropriate local action and planning priorities to address the gaps and challenges.

These activities succeeded in overcoming or at least lowering perceived barriers between different groups and brought actors into conversation with each other, drew on their shared informal knowledge of local health contexts, and provided opportunities to generate shared meanings about those contexts.

These ‘conversations’ have subsequently led to some specific health initiatives, such as continued action to share knowledge among groups and tackle environment health problems in certain communities. However, maintaining local area groups in all areas in the sub-district has proved difficult.

*The local government PHC facility manager ‘KPA process’ (developing local service improvement priorities)*

In 2010, the local government sub-district health manager introduced a new process to encourage pro-active planning and action by facility managers, involving: i) setting clear, locally appropriate objectives within the broad priorities specified in established health plans; ii) outlining activities, intended outcomes and monitoring and evaluation approaches; and iii) holding facility managers to account for implementing agreed actions.

Working with support, facility managers each developed their own KPAs and then presented them to the whole sub-district managerial team in late 2010. During the course of 2011 they periodically reported back on progress in implementing agreed actions and in late 2011 developed a new set of KPAs for 2012. In parallel, the routine PDR meeting between facility managers and their line managers in which facility challenges are discussed, was re-named and re-structured to allow a stronger regular focus on collectively considering how to address common PHC facility challenges, including sharing ‘best practices’ and success stories among these managers. Using existing language and the KPA terminology to introduce the new process, the sub-district manager, nonetheless, reframed this language to emphasize its developmental and sense-giving potential. She also role-modelled constructive accountability through creating a space to allow collective consideration of challenges and successes.

After two years, implementation is uneven, as follow-up and consistent documentation have been lacking. While some managers easily saw and acted on the opportunity to self-determine priorities, others will need more support to gain the confidence and skills to identify and act on local priorities.

Reflecting on our activities through theoretical lenses throws light on five possible elements of a ‘leadership of sensemaking’ for PHC strengthening. The importance of middle managers’ personal values as a foundation for other leadership action is the first element [14]. Leadership values and capabilities of particular relevance to sensemaking for PHC might include concern for the population being served and the broader social determinants of health; recognizing the potential in other people, for example by adopting a mentoring approach towards other staff; and being reflective and self-critical – willing to learn and change one’s own practices (Manager interview notes, 13th April 2012; 9th April 2013; 20th May 2013).

From this foundation, we have applied four other cross-cutting leadership practices in supporting PHC facility managers:

i. Nurturing the values and moral purpose of PHC staff

ii. Building relationships to support the development of shared meanings about change

iii. Instilling a culture of collective inquiry and mutual accountability within the sub-district

iv. Role modelling critical management practices and using language to signal new meanings

First, nurturing the values and moral purpose of PHC staff – encouraging facility managers, for example, ‘to dream’ about working differently (ISDMT meeting notes, 20th October 2010).

Re-orienting front line health staff towards a population health focus, encouraging them to take a pro-active role in managing services to meet community need, requires a “*real mind-shift for managers and staff*”*.* “*A community orientation has to become part of people’s way of being*” (ISDMT meeting notes, 19th Jan 2011), but some facility managers and staff do not currently have, and even resist, this orientation. The sub-district managers, therefore, constantly and consistently affirm the importance of patients and the broader population in all their engagements with staff – for example, encouraging facility managers to align broader goals and targets with local priorities, or to respond speedily to patient complaints.

Within the DIALHS collaboration, we also, more formally, initiated a collaborative community engagement process in 2011 to encourage conversation about local health needs and resources among different stakeholders in the sub-district (see Community profiling initiative). We expressly framed this activity within the context of the social determinants of health and allowed facility managers to think about the world outside their facilities. In implementing this process, sub-district managers noted that it was important to role model new mind-sets and use new language: “*whoever looks at us needs to know that as a team we are committed to the DHS and PHC, and building it with a population focus and orientation– and this is what we are working towards, this is what DIAHLS is supporting… It needs the full support of the ISDMT, every member needs to really believe in the process, to understand it and be committed to it. They need to talk positively about it when talking to facility managers and other staff, they need to take roles in making it happen and really support it.*” (ISDMT meeting notes, 19th Jan 2011).

The importance of such an approach is only affirmed by theory. “*Leaders must foster learning and values… They need a sense of optimism that can help the system deal with complexity, risk taking, and uncertainty. They need to help the system maintain a coherent identity*” [28]. Shared values and visions may, moreover, act as catalysts of change within a CAS, especially when they emerge through experience, providing the common energy that encourages and enables commitment to action across people within a system [14].

Second, in line with wider thinking [14], we have created spaces and processes where facility managers can be brought into relationship with each other, with colleagues in the sub-district and with other local actors, to share knowledge and ideas, challenge each other and learn from each other. The community profiling initiative, for example, initially comprised a process of sharing ideas and experience in drawing onto physical maps. Facility managers commented on the value of seeing the world through others’ eyes, realizing also how knowledgeable community members are, and on having opportunities to talk with other local actors outside the pressurized atmosphere of their facilities.

Several of the routine sub-district meetings have, furthermore, been adapted to provide opportunities to share and discuss experience about achievements, challenges, and priorities and to give space to developing team working among facility managers and with sub-district colleagues responsible for human resource management, supply management, and information systems. Meeting spaces also provide opportunities to develop new forms of accountability, to move away from the top-down approach perceived as checking progress towards targets and disciplining failure, towards a shared engagement about what enables and prevents progress, developing collective responses to tackling challenges: “*it’s not about holding people accountable, but providing a space to be supportive in holding them accountable*” (Researcher field notes, 6th June 2012; see also Key performance area process).

Within DIAHLS, we have also thought quite carefully about meeting practices that allow more active engagement and ownership by all those present, rather than primarily being spaces where information or instructions are transmitted from managers to staff. Rotating the task of chairing, using rounds to allow each person to make an input to the meeting, and asking challenging questions are, for example, ways of demonstrating equality, rather than reinforcing existing bureaucratic power balances, and of sharing experience to identify where support is needed. “The *basic assumption is that opening up the meetings in these ways makes them less intimidating or threatening, and allows better communication – a two-way flow of ideas, between sub-district and facility managers in particular, but also to contextualize information about new activities for other programmes and support staff, and so encourage greater understanding and ownership of the activities*” (Researcher field notes, 6th June 2012).

Third, through meetings and other routine activities the sub-district managers are also trying to instil a new culture of reflection and questioning – trying to encourage facility managers to ask ‘does it make sense, how must it be done, can it be done better?’ As one manager argued, “*We have to change the way we do things, and that means not accepting how things currently are.*” (Manager interview notes, 13th April 2012).

The ‘KPA process’, for example, specifically sought to respond to the negative perceptions around target setting by providing a space to allow facility managers to identify their own priorities whilst working within existing planning frameworks and job descriptions. The intentions were to encourage understanding of higher level strategic priorities, forward planning at facility level, engagement with data relevant in setting priorities, and to develop “*an attitude which looks at the underlying causes of challenges so that you can actually get to the systemic issues you need to change to improve the whole picture*” (Researcher field notes, 19th July 2012).

Finally, sub-district managers are role modelling more systematic approaches to management through their personal practices [22]. For example, in how they conduct staff appraisal discussions, being available on time for meetings and being respectful in their treatment of colleagues, as well as by coaching staff, running staff workshops in ‘difficult’ facilities and providing hands on support to weaker managers (Manager interview notes, 1st April 2011; 15th October 2012; 7th December 2012). The research team members have, meanwhile, sought to role model reflective practice through their research approach, for example, and in their approach to managing meetings.

We also all recognize the power of language, through which managers are “*able to articulate meanings, lend weight to collective action, and clarify the hoped for image of the organisation*” [45]. The quarterly PDR meeting for local government facility managers, for example, has been deliberately renamed the Management and Communication meeting as a response to the sense that the managers “*felt the name of the ‘PDR’ led people to worry, as they understood it to be essentially about criticizing them for not reaching their targets*” (Researcher field notes, 6th June 2012).

Ultimately, through these various practices, the sub-district managers are seeking “*to use the intangible in combination with the tangible in ways that keep an eye on the goal* [of PHC]*, but do things a bit differently – that’s bottom up power*” (Research meeting notes, 5th December 2012).

## Discussion

These Mitchell’s Plain experiences show how, despite individuals’ agreement with overall policy goals, efforts to strengthen PHC confront facility managers’ collective weakness to engage in pro-active, local-level problem solving in support of population health and equity goals. This reluctance reflects collective mind-sets of passivity and risk avoidance rooted in three key experiences: authoritarian cultures and histories; nearly two decades of centrally-driven policy and organisational change; and growing complexity in patient demands. These experiences, in turn, underpin vicious cycles of passivity, resistance to change, and further passivity, illuminating the ways in which, as Lipsky foresaw [29], sensemaking and the exercise of discretionary power are intertwined.

The experiences also suggest that beyond developing guiding visions about PHC strengthening, leadership for PHC must support facility managers to take ownership of these visions collectively. The visions must make sense to them if they are to incorporate them into their practices and so exercise their discretionary power in pursuit of PHC goals. Lipsky [29] identified, for example, the importance of supporting front line workers through “*ongoing processes of supportive criticism and inquiry. Built into every week of practice should be opportunities to review individual’s work, share criticisms, and seek a collective capacity to improve performance*”. CAS theory, meanwhile, notes that, to support system change, leaders must create the conditions for the emergence of such change – in particular, by encouraging the cycles of action, feedback and learning that empower system actors to think and work differently [45].

Mitchell’s Plain sub-district managers have, partly in collaboration with researchers within the DIAHLS project, initiated various activities intended to provide spaces of collective sensemaking to empower facility managers in these ways. Although these sensemaking efforts are still in their early days, the overall approach is affirmed by wider theory and empirical experience in two ways. First, in this broader literature, middle managers are recognized to play important and very particular roles as change intermediaries in organisational sensemaking, because they are positioned at the interface between an organisation’s senior managers and front line employees [36]. From this position, “…*interpretation is a key role. They need to ‘make sense’ upwards with senior managers, and laterally with peers and downwards with teams, to aid their interpretation of change intent and negotiate how change should be taken forwards*” [25,33,46]. Second, to support organizational sensemaking, the literature suggests that middle managers must reflect on their own perspectives and behaviours, thinking about how they will help others through change. They will need to create spaces for conversation, reflection, and dialogue, perhaps around planning, resource allocation, and monitoring processes – allowing colleagues to revisit their own mental models, understand those of others, and develop shared interpretations and meanings of change [25,28,29]. More concretely, and directly reflecting Mitchell’s Plain experience, the ‘Leadership to support implementation of nurse-led community health programmes’ section below outlines the leadership practices applied within a UK experience of organisational change around nurse-led community health programmes [47,48].

*Leadership to support implementation of nurse-led community health programmes [Source: 47]*

• *Created conditions for change through reflection, debate and challenge, workshops, skills audits, and education programmes.*

• Action learning to facilitate understanding of mental models and enable challenge to those models, leading to modification.

• *Worked with the ‘strange attractors’ (experiences or forces that attract the energies and commitment) that motivated practitioners (individual clients for some, whole communities for others).*

• Introduced new relationships that led to new ideas, emergent behaviours, and work patterns.

• Held multiple participatory events to encourage involvement, collaboration, and self-organisation.

• Established a few clear rules through discussion.

Nonetheless, we recognize that the sensemaking activities we have initiated will, inevitably, unfold in unpredictable ways over time. We do not expect that they will generate immediate and obvious change in sub-district performance, and instead we seek to encourage some change in meanings and practices that we hope will, over the longer-term, help embed a population health orientation within the local health system. Such leadership recognizes that “*the order in organizational life comes just as much from the subtle, the small, the relational, the oral, the particular and the momentary as from the conspicuous, the large, the substantive, the written, the general and the sustained*” [26].

Some indications of the positive potential of our sensemaking work lie in the perceptions of managers outside the sub-district. They report seeing a difference in the way things work in Mitchell’s Plain, in the willingness of staff to work together and tackle problems, and that they see the results in implementation of service delivery improvements. Inside the sub-district, some facility managers also report finding the KPA process helpful as it provided a sort of on-the-job training and supported local level decision making to tackle problems. There are also clear indications of stronger peer support among facility managers.

However, our experience only confirms another theoretical insight – that it takes significant energy and attention to prevent personal and system practices falling back into pre-established patterns [26]. Maintaining energy around the local area groups that developed through the community profiling, for example, has been a challenge, and facility managers have become dis-engaged from the activity (see *Community profiling initiative* above). Staff turnover also represents a significant challenge to institutionalizing new practices and meanings: for example, whilst the KPA process (see *Key performance area process* above) had some impact on some of those involved, newly appointed facility managers did not receive much orientation or support for their engagement in this process and so were not really sure what they were doing.

For middle managers, meanwhile, the Mitchell’s Plain experience indicates that the challenges of leadership include the ways in which their sensemaking efforts are filtered through other colleagues who may not adopt similar approaches or who may themselves be threatened by new approaches and ideas. In the face of facility managers’ apparent passivity and lack of confidence, it may also be easy to slip back into micro-management; and, in the face of external demands, it is hard to maintain positive role modelling. To support organizational change middle managers not only need negotiation, persuasion and advocacy skills, but also, support from higher-level managers – such as the time and flexibility to sustain sensemaking engagement with those on the front line of organizational change/policy implementation [22,36,46], consistent and positive messages about new activities from higher levels of the system, and a willingness to allow experimentation to fine tune these activities.

## Conclusions

Although exploratory, this analysis adds to the still limited body of work examining health system complexity [49], the influence of actors over policy implementation [50], and the leadership needed to support system-wide reforms in pursuit of population health and equity goals [12]. Indeed, we believe that this is the first paper specifically to begin to consider how sensemaking and discretionary power work together in challenging or supporting PHC re-orientation within a middle-income country health system, or to consider what a leadership of sensemaking for PHC entails. It has been made possible by the long-term and collaborative nature of the DIAHLS project, which supports the co-production of knowledge about the inner workings of the Mitchell’s Plain health system. Building on experience so far, we will continue to work together to strengthen, and track over time, our efforts to support PHC performance improvement.

Our core argument is that:

i. The system-wide population health re-orientation needed to sustain PHC in South Africa will only become a lived reality when the front line staff who work at the health system’s interface with the population bring it alive within their everyday routines and practices;

ii. These agents’ sensemaking capabilities mean centrally-directed initiatives intended to strengthen PHC are re-interpreted as implemented, with unexpected consequences that can include resistance to centrally-led activities (i.e., exercising their discretionary power in ways that thwart such initiatives);

iii. New forms of middle manager (and wider) leadership are required to nurture collective sensemaking around PHC goals and empower front line health staff to take ownership of these goals, and so exercise their discretionary power in their pursuit;

iv. Mind-set changes, focused on concern for the population being served, the broader social determinants of health, and a willingness to act, are likely to be the fundamental basis for strengthening and sustaining PHC.

In a complex adaptive system “*…organisational change is not management induced. Instead, organizational change is emergent change laid down by choices made on the front line*” [26]. Nurturing such change at the front line of the health system requires, therefore, new forms of leadership that enable sensemaking in support of change and unleash the collective power distributed across the system towards shared goals.

## Endnotes

^a^The sub district population is around 510,000 and so is large in comparison to the WHO definition of a health district. In South Africa, the nine provinces each have constitutional authority for managing health services in their area and implementing health policy, with local governments having concurrent responsibility for managing aspects of primary health care.

^b^Since 1994, structural, management, and service delivery developments within the Western Cape province health system have been guided by three inter-linked health policy documents: the 1995 *Provincial Health Plan*, the 2005 *Comprehensive Service Plan* (2005), and, most recently, the *Healthcare 2030* policy document (available at http://www.westerncape.gov.za). Working within national policy frameworks, they provide a guiding vision for organizational change of a health system oriented towards population health needs and founded on a strong district health system.

## Abbreviations

CAS: Complex adaptive systems; CoCT: City of Cape Town; DHS: District health system; DIALHS: District Innovation and Action Learning for Health System Development; ISDMT: Integrated sub-district team; KPA: Key performance area; MDHS: Metro District Health System; PDR: Plan, do, review; PHC: Primary health care; SLB: Street level bureaucracy; TB: Tuberculosis.

## Competing interests

The authors declare that they have no competing interests.

## Authors’ contributions

LG co-led the conceptualization of the paper and related data analysis, wrote the first draft of the paper, and revised it with comments from other authors. SE contributed to the paper’s conceptualization and data analysis, and commented on drafts of the paper. PO contributed to the paper’s conceptualization and data analysis, and commented on drafts of the paper. UL co-led the conceptualization of the paper and related data analysis, contributed to the first draft of the paper and commented on subsequent drafts. All authors read and approved the final manuscript.
